# First nationwide investigation of *Cryptosporidium* species and *gp60* subtypes in dairy cattle in Cyprus

**DOI:** 10.1016/j.crpvbd.2025.100326

**Published:** 2025-10-13

**Authors:** Daphne E. Mavrides, Maria Liapi, Stavros Malas, Anastasios D. Tsaousis, Eleni Gentekaki

**Affiliations:** a Department of Veterinary Medicine, University of Nicosia School of Veterinary Medicine, 2414, Nicosia, Cyprus; b Department of Basic and Clinical Sciences, University of Nicosia Medical School, 2408, Nicosia, Cyprus; c Veterinary Services of Cyprus, Nicosia, Cyprus; d The Cyprus Institute, Nicosia, Cyprus; e Laboratory of Molecular and Evolutionary Parasitology, School of Natural Sciences, University of Kent, Canterbury, CT2 7NJ, UK

**Keywords:** *Cryptosporidium*, Dairy cattle, Zoonosis, Subtyping, Cyprus, Infectious diseases, Agriculture

## Abstract

*Cryptosporidium* spp. are globally important enteric pathogens in livestock and a leading cause of neonatal calf diarrhoea, with zoonotic potential. This study presents the first nationwide molecular survey of *Cryptosporidium* spp. and *C. parvum gp60* subtypes in dairy cattle in Cyprus. A total of 517 faecal samples were collected from dams (*n* = 256) and their 3–7-day-old calves (*n* = 261) across 18 farms in five districts. Samples were screened by nested PCR and sequencing of the SSU rRNA and *gp60* genes. Overall, *Cryptosporidium* spp. prevalence was 28.2%, with higher rates in calves (39.5%) than in dams (16.8%). *Cryptosporidium parvum* was the dominant species (85.6%), followed by *C. bovis* (8.2%), *C. andersoni* (4.8%), and *C. ryanae* (0.7%). Eight *C. parvum gp60* subtypes were identified, six from family IIa and two from IId, the latter reported for the first time in Cypriot cattle. Subtypes IIaA14G1R1 and IIdA16G1 were strongly associated with severe diarrhoea, while IIaA17G2R1 predominated in asymptomatic calves. Several identified subtypes have been implicated in human outbreaks globally. Cyprus’s confined livestock population and strategic location at the crossroads of Europe, Asia, and Africa provide a unique context for understanding *Cryptosporidium* transmission. This study establishes essential baseline data on the prevalence, molecular diversity, and zoonotic potential of *Cryptosporidium* in an island setting. The findings underscore the need for integrated surveillance and One Health strategies to control transmission, protect public health, and monitor the emergence of high-risk subtypes locally and beyond.

## Highlights

• First nationwide study of *Cryptosporidium* spp. in dairy cattle in Cyprus.
• *Cryptosporidium* spp. detected in 28.2 % of samples from cattle, 39.5 % of calves and 16.8 % of dams.
• Eight *C. parvum gp60* subtypes found; first description of family IId in Cyprus.
• Only IIaA14G1R1 and IIaA15G2R1 overlapped with those previously reported in Cyprus.
• IIaA14G1R1 and IIdA16G1 associated with diarrhoea, IIaA17G2R1 with no symptoms.

## Graphical abstract

**Figure:**
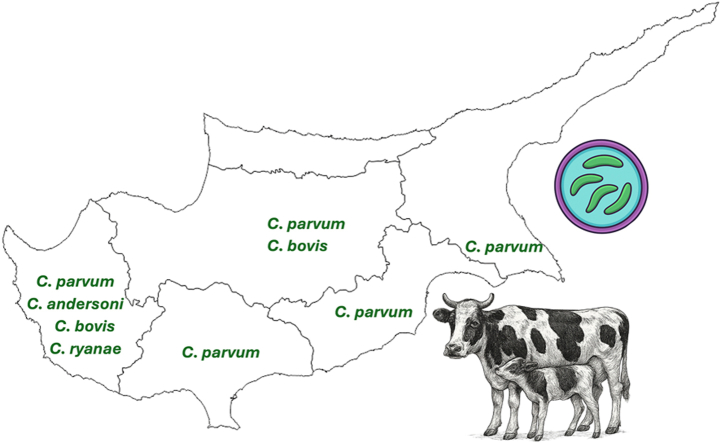

## 1 Introduction

*Cryptosporidium* spp. are apicomplexan parasites and one of the most common causes of enteric disease in young ruminant livestock, particularly in neonatal calves (Suler et al., 2016; Arsenopoulos et al., 2017; Thomson et al., 2017; Hoque et al., 2022), with four species being commonly found in cattle: *Cryptosporidium andersoni*, *Cryptosporidium bovis*, *Cryptosporidium parvum*, and *Cryptosporidium ryanae. Cryptosporidium bovis* and *C. ryanae* have been predominantly detected in post-weaned calves, *C. andersoni* in adult cattle, whilst *C. parvum* is most abundant in pre-weaned calves (Santín et al., 2004, 2008; Díaz et al., 2021).

Of the species described, *C. parvum* is zoonotic and the most pathogenic, although *C. andersoni* and *C. bovis* are also regarded as potentially zoonotic, with sporadic human infections by *C. bovis* reported globally (Khan et al., 2010). *Cryptosporidium parvum* causes profuse, life-threatening diarrhoea in calves and is also highly contagious to other livestock, including lambs and kids, as well as humans. Regarding the latter, cryptosporidiosis can cause severe gastrointestinal disease, with children and immunocompromised individuals being particularly vulnerable (Dixon, 2014; Thomson et al., 2017). The large capacity of *C. parvum* to reproduce and disseminate a high number of environmentally resilient oocysts (i.e. 10^7^ oocysts/gram), which are also resistant to most chemical disinfectants, accounts for its high contagiousness (de Graaf et al., 1999; Nydam et al., 2001; Chalmers, 2014; Suler et al., 2016; Bordes et al., 2020). Pre-weaned calves are considered reservoirs accounting for human infections and environmental contamination (Gong et al., 2017; Hatam-Nahavandi et al., 2019). In line with this, livestock manure is assumed to be the primary environmental source harbouring these oocysts in Europe and North America, though this has yet to be validated with hard data or modelling (Vermeulen et al., 2019). This introduces a public health concern where contaminated drinking and recreational waters can reach humans (Bordes et al., 2020). Zoonotic transmission typically occurs *via* the faecal-oral route and usually through waterborne outbreaks with the potential to affect many people simultaneously, but food-borne transmission is also possible (Chalmers, 2014; Dixon, 2014; Suler et al., 2016). *Cryptosporidium parvum* also causes significant economic losses to the agricultural sector (de Graaf et al., 1999; Suler et al., 2016; Shaw et al., 2020). This can amount to €40 per diarrhoeic calf due to mortality losses, reduced productivity of affected calves that have survived infections, health expenditures and additional labour costs for the care and management of ill calves (Roblin et al., 2023).

Genotyping and subtyping are key to understanding the molecular epidemiology of *Cryptosporidium* spp. While species identification is commonly performed using PCR targeting the SSU rRNA gene, subtyping within species, particularly for *C. parvum*, relies on sequencing a fragment of the highly polymorphic 60 kDa glycoprotein gene (*gp60*) (Xiao and Feng, 2017). The *gp60* gene encodes an immunodominant surface glycoprotein involved in host cell invasion, contributing to host pathogenicity (Li et al., 2024). Its high degree of sequence variation, including polymorphisms and mini- and microsatellite repeats, has made it a valuable target for subtyping and has formed the basis for the current *gp60* nomenclature system (Robinson et al., 2025).

There have been numerous studies concentrating on *Cryptosporidium* spp. and *C. parvum* subtype identification in cattle worldwide (Becher et al., 2004; Alves et al., 2006; Broglia et al., 2008; Brook et al., 2009; Amer et al., 2010; Baroudi et al., 2017; Avendaño et al., 2018; Ayres Hutter et al., 2020). However, regions remain where knowledge of the parasites’ epidemiology is still lacking. Hence, this study focuses on Cyprus, an island in South Europe close to Africa and the Middle East. There is limited information on the epidemiology of *Cryptosporidium* spp. in Cyprus, with relatively few studies conducted to date. Existing research has focused mainly on goats and sheep, as well as some wildlife species such as mouflon, reptiles and birds (Giadinis et al., 2012; Schou et al., 2022; Hasapis et al., 2023, 2024). Only one molecular study has investigated *Cryptosporidium* spp. in cattle. This previous, small-scale study focusing primarily on dairy calves found a high occurrence of *Cryptosporidium* spp. (43.8%) and identified three species: *C. parvum*, *C. bovis*, and *C. ryanae*, with *C. parvum* being the most prevalent. Subtyping at the *gp60* locus revealed the presence of subtypes IIaA12G1R1, IIaA14G1R1, IIaA15G1R1, IIaA15G2R1 and IIaA18G2R1, marking the first report of zoonotic *C. parvum* subtypes in cattle in Cyprus (Hoque et al., 2022). Herein, we expand this investigation further to include additional districts of Cyprus and a larger sample size that includes both calves and their mothers, hence considering dam-to-calf transmission and geographical distribution. This nationwide study enriches our understanding of *Cryptosporidium* spp. epidemiology on the island. It can be used as a platform for future studies on the incidence, source of infection and transmission dynamics of the organism.

## 2 Materials and methods

### 2.1 Sampling sites and collection

A total of 37 cattle farms, selected from a national registry of 358, were included in the study, as previously described (Mavrides et al., 2024). Farm owners were contacted by telephone to obtain consent for participation. The inclusion criteria classified dairy farms located across diverse regions and altitudes of the island, each maintaining more than 100 Holstein cows. These 37 farms ranged from 122 to 1297 cows in size, with a median of 524. The farms operated under intensive management systems, where cows were housed year-round and provided with supplemental feeding. The authors were unaware of any reports of cryptosporidiosis on the participating farms prior to sampling. Faecal samples were collected from both dams and their newborn calves within three to seven days of birth from each farm. The seven-day cut-off in age was decided based on the practice of several farms to separate calves from the dams by that time. Henceforth, it would not be possible to track calves back to their mothers. A total of 519 faecal samples were collected directly from the rectum of 257 dams and their corresponding 262 calves, 51% of which were female and 49% male. Of these, 220 samples were collected from Nicosia, 125 from Paphos, 120 from Limassol, 28 from Larnaca and 26 from Ammochostos. Samples were collected at random and categorised as either normal or diarrhoeic. Diarrhoeic samples were further classified according to severity using the ‘Calf Health Scorer’ system developed by the University of Wisconsin School of Veterinary Medicine (https://www.vetmed.wisc.edu/), with grades of one (semi-formed), two (runny), or three (watery). All samples were collected in sterile tubes and immediately transported to the laboratory, where they were stored at −80°C until further analysis.

### 2.2 DNA extraction

Samples underwent DNA extraction within three months after collection. DNA was extracted using 200 mg of each stool sample with the PureLink™ Microbiome DNA Purification Kit (Thermo Fisher Scientific, Carlsbad, CA, USA) according to the manufacturer’s instructions with modifications. Specifically, 650 μl of Lysis Buffer was added to each sample instead of 600 μl. Following the addition of 100 μl of Lysis Enhancer, the samples were incubated at 95 °C for 15 min rather than 65 °C for 10 min and then homogenised by bead beating for 13 min. The samples were incubated at 4 °C for 10 min following the addition of 250 μl of Cleanup Buffer. Finally, 50 μl instead of 100 μl of Elution Buffer was added to the samples before incubating at room temperature for 5 min and then centrifuging. Extracted DNA was stored at −80 °C before PCR amplification was carried out.

### 2.3 Initial detection (screening) using SSU rRNA-PCR

Genomic DNA from stool samples was screened for *Cryptosporidium* spp. using nested PCR amplification of a 631-bp region of the small subunit ribosomal RNA (SSU rRNA) with barcoding primers (Ziegler et al., 2007; Pinto et al., 2021), performed on a MJ Research PTC-200 Thermal Cycler Dual 48 thermocycler. A negative control containing no faecal DNA and a positive control containing genomic DNA from a pure culture of *C. parvum* were used in both reactions and every PCR experiment. The primary reaction was conducted using the primers CRY_SSU_F1 (5′-GAT TAA GCC ATG CAT GTC TAA-3′) and CRY_SSU_R1 (5′-TTC CAT GCT GGA GTA TTC AAG-3′) (product size: 723 bp) and the secondary reaction of the nested PCR was conducted with the forward primer CRY_SSU_F2 (5′-CAG TTA TAG TTT ACT TGA TAA TC-3′) and the reverse primer CRY_SSU_R2 (5′-CCT GCT TTA AGC ACT CTA ATT TTC-3′) (Ziegler et al., 2007). Each PCR reaction mixture included 12.5 μl of PCRBIO Taq Mix Red, 1 μl of 10 μM forward primer, 1 μl of 10 μM reverse primer, 1 μl of template DNA (either sample DNA or control, concentration 50–100 ng/μl) and 9.5 μl of sterile Milli-Q water. Cycling conditions of the first reaction were 2 min at 94 °C, followed by 24 cycles of 50 s at 94 °C, 50 s at 53 °C, and 1 min at 72 °C. The final extension was 10 min at 72 °C. Cycling conditions of the second reaction were 2 min at 94 °C, followed by 30 cycles of 50 s at 94 °C, 30 s at 56 °C, and 1 min at 72 °C. The final extension was 10 min at 72 °C.

### 2.4 Genotyping using *gp60*-PCR

DNA samples that yielded an amplicon of the expected size in the SSU rRNA PCR underwent PCR amplification of the 60 kDa glycoprotein (*gp60*) gene using a nested PCR protocol to identify the subtype of *C. parvum* if present (Alves et al., 2003; Pinto et al., 2021). A positive and negative control was used in both reactions as described for the PCR amplification of the SSU rRNA. The primary reaction was conducted using the primers AL3531 (5′-ATA GTC TCC GCT GTA TTC-3′) and AL3535 (5′-GGA AGG AAC GAT GTA TCT-3′) (product size: 1000 bp) and the secondary reaction of the nested PCR was conducted using the forward primer AL3532 (5′-TCC GCT GTA TTC TCA GCC-3′) and the reverse primer AL3534 (5′-GCA GAG GAA CCA GCA TC-3′) (∼850 bp) (Alves et al., 2003). Each PCR reaction mixture included 15 μl of PCRBIO Taq Mix Red, 0.6 μl of 10 μM forward primer, 0.6 μl of 10 μM reverse primer, 2 μl of template DNA (either sample DNA or control, concentration 50–100 ng/μl) and 11.8 μl of sterile Milli-Q water. Cycling conditions of the first reaction were 3 min at 94 °C, followed by 35 cycles of 45 s at 94 °C, 45 s at 50 °C, and 1 min at 72 °C. The final extension was 7 min at 72 °C. Cycling conditions of the second reaction were 3 min at 94 °C, followed by 35 cycles of 45 s at 94 °C, 45 s at 50 °C, and 1 min at 72 °C. The final extension was 7 min at 72 °C.

### 2.5 Gel electrophoresis, PCR amplicon purification and sequencing

Agarose gel electrophoresis was used to separate the PCR products. Two percent agarose gels were cast with the use of TAE buffer, agarose powder and 2 μl ethidium bromide for staining. After loading the samples and ladder (100-bp DNA ladder) into each well, the gels were run at 46 V until the products separated. A gel-doc system (Bio-Rad Laboratories, Hercules, CA, USA) was used to visualise the gels. Amplicons within the regions of interest were cut and purified using the GeneJET Gel Extraction Kit (Thermo Fisher Scientific, Carlsbad, CA, USA). The purified products were sent for Sanger sequencing at an external provider (Eurofins Genomics, Wolverhampton, UK) using the primers used for the nested reaction.

### 2.6 Sequence analysis

Following Sanger sequencing, chromatograms were visually inspected, and ambiguous bases were trimmed from both ends. To identify organisms at the species level, SSU rRNA sequences were used as queries in BLAST searches against the GenBank nucleotide database. The *gp60* gene sequences were aligned and compared with reference sequences in GenBank. Subtypes were determined using established standard terminology (Xiao and Feng, 2017; Chalmers et al., 2019). Furthermore, sequences from each subtype were aligned with MUSCLE v5 to examine intra-subtype polymorphisms.

### 2.7 Statistical analysis

Global chi-square analyses were run to test overall associations, pairwise chi-square tests for detailed comparisons and odds ratios with Fisher’s exact tests to estimate the strength of associations. Global chi-square tests of independence were used to assess differences in: diarrhoea grade distributions between *C. parvum-*positive and -negative animals; diarrhoea status (no diarrhoea, Grades 1–3) among subtypes; diarrhoea grades across districts; and subtype distributions among districts. For the latter analysis, because certain cells contained zeros (no detection of a specific subtype in a district), expected counts were examined to ensure validity of the test assumptions. Pairwise chi-square tests were used to identify grade-specific differences (Grade 1 *vs* 2, Grade 1 *vs* 3, Grade 2 *vs* 3), to compare diarrhoea status among subtypes and to compare diarrhoea status patterns between districts. Odds ratios with Fisher’s exact test were estimated to assess associations between specific diarrhoea grades, to compare the likelihood of diarrhoea presence between subtypes and to compare overall proportions of diarrhoeic animals between districts. Statistical significance for chi-square and Fisher’s exact tests was defined at *P* < 0.05.

## 3 Results

Sampling took place over a one-year period, starting in September 2021 and ending in September 2022. The farms are located in Nicosia (6/18), Larnaca (4/18), Limassol (3/18), Ammochostos (2/18) and Paphos (3/18), reflecting the relative proportions of farms per district (Table 1).

**Table 1:** Presence of *Cryptosporidium* spp. in cattle farms in Cyprus.

Farms	No. of samples	No. of positive samples	Positive samples for *C. parvum* (SSU & *gp60*) (A)	Positive samples for *C. parvum* (SSU only) (B)	Positive samples for *C. parvum* (*gp60* only) (C)	Total *C. parvum* positive samples (A + B + C)	*C. bovis*	*C. andersoni*	*C. ryanae*	*Cryptosporidium* spp.
Nicosia 1	32	8	3	4	1	8				
Nicosia 2	33	6	2	1	2	5				1
Nicosia 3	40	2	1	1	0	2				
Nicosia 4	41	6	2	2	0	4	2			
Nicosia 5	41	13	9	2	2	13				
Nicosia 6	33	11	11	0	0	11				
Larnaca 1	4	1	0	1	0	1				
Larnaca 2	10	6	1	4	1	6				
Larnaca 3	10	1	0	1	0	1				
Larnaca 4	4	0	0	0	0	0				
Limassol 1	40	17	13	4	0	17				
Limassol 2	41	22	12	6	4	22				
Limassol 3	39	18	12	3	3	18				
Ammochostos 1	20	8	2	1	5	8				
Ammochostos 2	6	0	0	0	0	0				
Paphos 1	42	27	7	1	8	16	4	6	1	
Paphos 2	40	11	4	1	3	8	3			
Paphos 3	41	23	9	5	5	19	3	1		
Total	517	180	88	37	34	159	12	7	1	1

### 3.1 Detection of *Cryptosporidium* spp. infections

*Cryptosporidium* spp. were present in 16 of the 18 farms sampled (Table 1), and occurrence varied between farms from 5.0% to 50.0%. The distribution of the samples positive for *Cryptosporidium* spp. by district was as follows: 41.7% (50/120) of the samples from Limassol; 36.6% (45/123) from Paphos; 25.0% (7/28) from Larnaca; 18.6% (41/220) from Nicosia; and 11.5% (3/26) from Ammochostos (Table 2).

**Table 2:** Positivity rates of *Cryptosporidium* spp. (SSU) and *C. parvum* (*gp60*) depicted for dams and calves separately and overall, from cattle farms in all districts of Cyprus.

District (#farms)	Samples overall	SSU overall (%)	*gp60* overall (%)	Samples dams	SSU dams (%)	*gp6*0 dams (%)	Samples calves	SSU calves (%)	*gp60* calves (%)
Nicosia (6)	220	41 (18.6)	33 (15.0)	108	6 (5.6)	0 (0)	112	35 (31.3)	33 (29.5)
Larnaca (4)	28	7 (25.0)	2 (7.1)	14	4 (28.6)	0 (0)	14	3 (21.4)	2 (14.3)
Limassol (3)	120	50 (41.7)	44 (36.7)	60	14 (23.3)	10 (16.7)	60	36 (60.0)	34 (56.7)
Ammochostos (2)	26	3 (11.5)	7 (26.9)	13	1 (7.7)	2 (15.4)	13	2 (15.4)	5 (38.5)
Paphos (3)	123	45 (36.6)	36 (29.3)	61	18 (29.5)	17 (27.9)	62	27 (43.5)	19 (30.6)
Total (18)	517	146 (28.2)	122 (23.6)	256	43 (16.8)	29 (11.3)	261	103 (39.5)	93 (35.6)

Amplification and sequencing of the SSU rRNA gene showed a *Cryptosporidium* spp. occurrence of 28.2% overall (146/517), 16.8% in dams (43/256) and 39.5% in calves (103/261) (Table 2). Sequencing of two samples was not successful, and they were removed from the sampling pool.

**Fig. 1:**
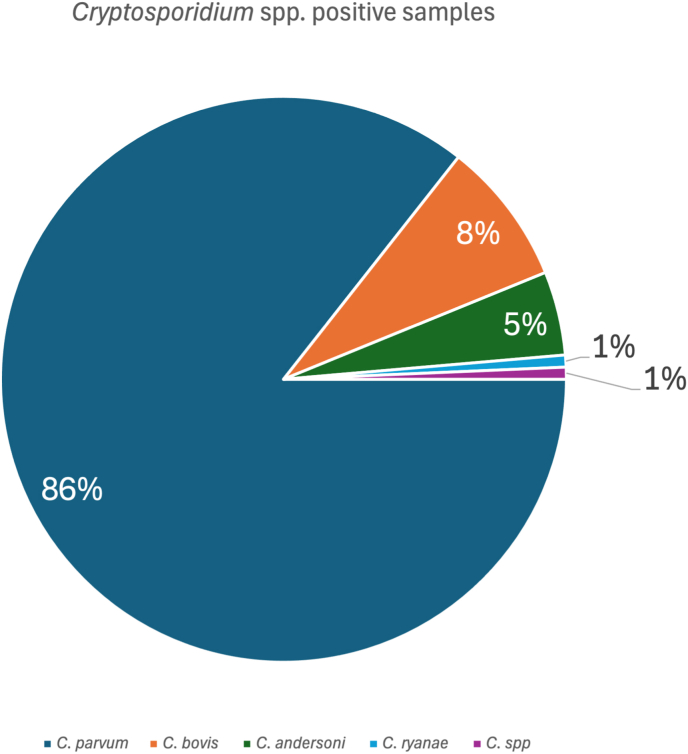
Distribution of *Cryptosporidium* species detected in cattle in Cyprus. Proportion of *Cryptosporidium* species identified among positive dairy cow stool samples (*n* = 146). *Cryptosporidium parvum* was the most abundant species (86%), followed by *C. bovis* (8%), *C. andersoni* (5%), *C. ryanae* (1%), and *Cryptosporidium* spp. (1%).

Of the *Cryptosporidium* spp.-positive samples, 85.6% (125/146) were identified as *C. parvum*. Good quality SSU rRNA sequences were not obtained for 13 PCR-positive samples. However, *C. parvum* identity was confirmed for five of these samples through *gp60* sequencing and the remaining eight were regarded as negative. *Cryptosporidium parvum* was present in all *Cryptosporidium* spp.-positive farms. The second most common species present was *C. bovis* at 8.2% (12/146 in four farms), followed by *C. andersoni* at 4.8% (7/146 in two farms) (Fig. 1). Only one sample positive for *C. ryanae* was detected. Both *C. andersoni* and *C. ryanae* were only detected in Paphos (Table 1). In addition, there were 10 samples with potential co-infections, 5 with *C. bovis* + *C. parvum* and 5 with *C. andersoni* + *C. parvum*, all originating from the Paphos district. These were identified as non-*C. parvum* species based on SSU rRNA gene sequencing, while the *gp60* locus from the same sample was successfully amplified and subtyped as *C. parvum*.

### 3.2 Diarrhoeic samples and *Cryptosporidium* spp. infections

**Fig. 2:**
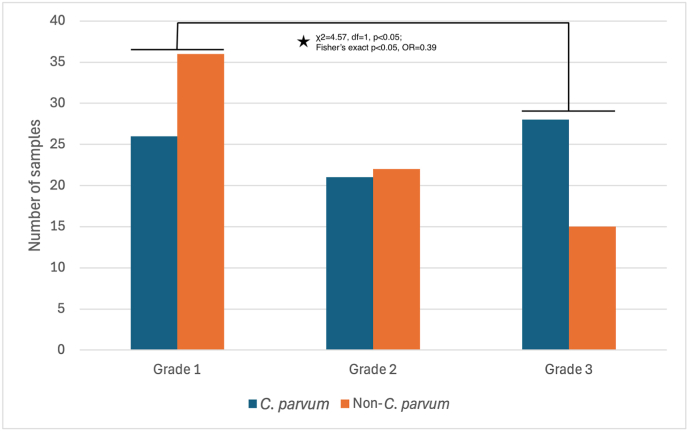
Distribution of *Cryptosporidium parvum* cases across diarrhoea grades. Number of positive (*blue*) and negative (*orange*) samples for *C. parvum* among calves aged 3–7 days with diarrhoea Grades 1–3. Non-*C. parvum* cases were more frequent in calves with Grade 1 diarrhoea, while *C. parvum* predominated in Grade 3 cases (severe diarrhoea). Statistical testing confirmed a significant association between the two grades (*χ*^2^ = 4.57, *df* = 1, *P* < 0.05; Fisher’s exact test, *P* < 0.05, OR = 0.39).

Of the 517 samples, 148 were diarrhoeic (28.6%, 148/517), most of which were from calves (95.3%, 141/148). Sixty-two (41.9%) of the diarrhoeic samples were marked as Grade 1, 43 (29.1%) as Grade 2, and 43 (29.1%) as Grade 3. Twenty-two (34.5%) samples of Grade 1, 16 (37.2%) samples of Grade 2 and 26 (60.5%) samples of Grade 3 were positive for *C. parvum* (*gp60*). Of the samples positive for *C. parvum* (based on SSU and *gp60* genes), 16.4% had Grade 1 (26/159), 13.2% had Grade 2 (21/159) and 17.6% had Grade 3 diarrhoea (28/159) (Fig. 2). The global chi-square test of independence indicated no association between diarrhoea grade and *C. parvum* presence (*χ*^2^ = 5.54, *df =* 2, *P >* 0.05). Pairwise comparisons showed a significant difference between Grade 1 and Grade 3, with animals having Grade 1 diarrhoea being significantly less likely to have *C. parvum* than those having Grade 3 (*χ*^2^ = 4.57, *df =* 1, *P* < 0.05; Fisher’s exact test, *P* < 0.05, OR = 0.39). Comparisons between Grade 1 and Grade 2 and between Grade 2 and Grade 3 diarrhoeic animals were not significant. Of the *Cryptosporidium* spp.-positive samples, 49.3% were diarrhoeic (72/146). Sixty-seven out of 220 (30.5%) samples from Nicosia were diarrhoeic, 29.2% (35/120) from Limassol, 28.6% (8/28) from Larnaca, 26.0% (32/123) from Paphos, and 23.1% (6/26) from Ammochostos. District level analyses were not significant except for the pairwise 2 × 4 chi-square test of diarrhoea grade distribution between Limassol and Paphos (*χ*^2^ = 9.49, *df* = 3, *P* < 0.05). The number of diarrhoeic samples per farm varied from 0 to 80%, with a mean of 31.7% for all farms. Of the *Cryptosporidium* spp.-positive samples that were diarrhoeic (49.3%, 72/146), 86.1% were positive for *C. parvum* (62/72), 12.5% for *C. bovis* (9/72) and 1.4% for *C. andersoni* (1/72), and all originated from calves. Of the *Cryptosporidium* spp.-positive samples that were not diarrhoeic (50.7%, 74/146), 85.1% were positive for *C. parvum* (63/74), 8.1% for *C. andersoni* (6/74), 4.1% for *C. bovis* (3/74), 1.4% for *C. ryanae* (1/74) and 1.4% for *Cryptosporidium* spp. (1/74). Twenty-seven of the *C. parvum-*positive non-diarrhoeic samples were from calves, and 36 were from dams. All of the *C. andersoni* and *Cryptosporidium* spp.-positive non-diarrhoeic samples were from dams, and all of the *C. bovis-* and *C. ryanae-*positive non-diarrhoeic samples were from calves (Table 2). Diarrhoea was present in 49.6% of *C. parvum*-positive samples (62/125), 75.0% of *C. bovis*-positive samples (9/12), and 14.3% of *C. andersoni*-positive samples (1/7). Of the diarrhoeic animals positive for *C. parvum*, 32.3% had Grade 1 (20/62), 24.2% had Grade 2 (15/62), and 43.5% had Grade 3 diarrhoea (27/62).

### 3.3 Age and *Cryptosporidium* spp. infections

**Fig. 3:**
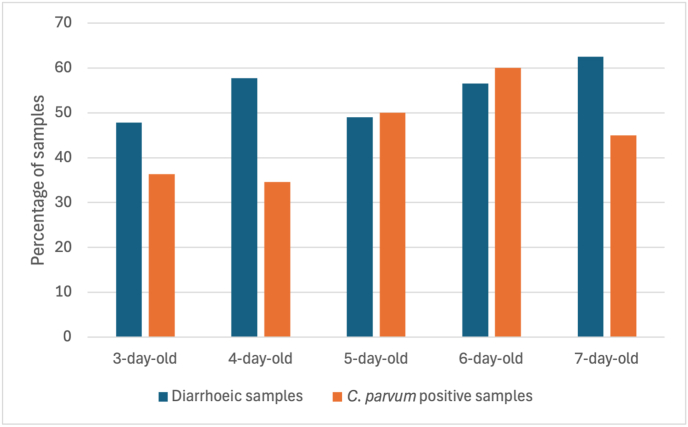
Cross-sectional distribution of diarrhoea and *Cryptosporidium parvum* positivity across calf age groups. Percentage of diarrhoeic samples (*blue*) and *C. parvum-*positive samples (*orange*) among calves aged 3–7 days. The highest proportion of diarrhoea (63%) was observed in the 7-day-old group of calves, while the highest *C. parvum* positivity (60%) occurred at the 6-day-old group.

*Cryptosporidium parvum* was predominantly detected in calves (71.2%, 89/125), *C. bovis* was only detected in calves (12 samples), *C. andersoni* was predominantly detected in adult cows (85.7%, 6/7), while the single *C. ryanae* was identified in a calf. Twenty out of the 32 7-days-old calves (62.4%) were diarrhoeic, followed by 57.8% (52/90) at 4-day-old, 56.5% (26/46) at 6-day-old, 49.1% (26/53) at 5-day-old, 47.8% (11/23) at 3-day-old, 28.6% (2/7) at 2-day-old and 0% (0/4) at 1-day-old (Fig. 3). More specifically, 57.7% (15/25) of the *C. parvum-*positive samples from 6-day-old calves were diarrhoeic, 50.0% from both 5-day-old and 2-day-old (13/26 and 1/2, respectively), 45.0% (9/20) from 7-day-old, 36.4% (4/11) from 3-day-old, 34.6% (18/52) from 4-day-old and 0 % (0/4) from 1-day-old (Fig. 3). For *C. bovis-*positive samples, 10.0% (2/20) of samples from 7-day-old calves were diarrhoeic, 9.1% (1/11) from 3-day-old calves, 7.7% from both 4-day-old and 5-day-old calves (4/52 and 2/26, respectively) and 0% from both 1-day-old and 2-day-old calves (0/4 and 0/2, respectively).

### 3.4 Subtyping of *Cryptosporidium parvum*

All the SSU rRNA PCR-positive samples were screened for *C. parvum* by PCR amplification of the *gp60* gene and subsequently sequenced. Eighty-eight of the SSU rRNA *C. parvum* sequence-positive samples were successfully amplified for the *gp60* gene and sequenced, 37 SSU rRNA *C. parvum* sequence-positive samples were not successfully amplified for the *gp60* gene, and 34 SSU rRNA PCR-positive samples that were unsuccessfully sequenced for SSU rRNA were successfully amplified for the *gp60* gene and sequenced. In total, 122 samples were amplified and sequenced for the *gp60* gene.

Sequence analysis revealed the presence of eight subtypes, six belonging to the IIa family (IIaA13G2R1, IIaA14G1R1, IIaA15G2R1, IIaA16G1R1, IIaA16G2R1, and IIaA17G2R1) and two to the IId family (IIdA16G1 and IIdA20G1) (Table 3). The most abundant subtype was IIaA17G2R1 (41.2%, 49/119) followed by IIaA14G1R1 (38.7%, 46/119), IIdA16G1 (10.1%, 12/119), IIdA20G1 (4.2%, 5/119), IIaA16G1R1 (2.5%, 3/119), IIaA15G2R1 (1.7%, 2/119), IIaA13G2R1 (0.8%, 1/119), and IIaA16G2R1 (0.8%, 1/119) (Table 3). Alignment of the individual subtypes revealed a few polymorphisms, mainly in the gp40 region, but no consistent pattern was detected. Three *gp60*-positive samples could not be subtyped due to unclear sequence chromatograms.

**Table 3:** Presence of *C. parvum gp60* subtypes from cattle farms in Cyprus.

Farms	IIaA13G2R1	IIaA14G1R1	IIaA15G2R1	IIaA16G1R1	IIaA16G2R1	IIaA17G2R1	IIdA16G1	IIdA20G1
Nicosia 1		4 (4C, 3D)						
Nicosia 2		4 (4C, 3D)						
Nicosia 3		1 (1C, 1D)						
Nicosia 4		2 (2C, 2D)						
Nicosia 5		11 (11C, 10D)						
Nicosia 6		10 (10C, 7D)						
Larnaca 1								
Larnaca 2		2 (2C, 1D)						
Larnaca 3								
Larnaca 4								
Limassol 1		7 (7C, 5D)				3 (3C, 2D)		3 (3C, 2D)
Limassol 2						7 (5M, 2C, 1D)	9 (9C, 8D)	
Limassol 3			1 (1C, 1D)			11 (5M, 6C, 1D)	3 (3C, 2D)	
Ammochostos 1				3 (3C, 2D)		3 (2M, 1C)		
Ammochostos 2								
Paphos 1			1 (1M)		1 (1C)	11 (6M, 5C, 3D)		2 (2C)
Paphos 2						7 (3M, 4C, 3D)		
Paphos 3	1 (1M)	5 (4C, 4D, 1M)				7 (5M, 2C, 2D)		
Total	1	46	2	3	1	49	12	5

*Abbreviations*: M, dams; C, calves; D, diarrhoeic.

**Fig. 4:**
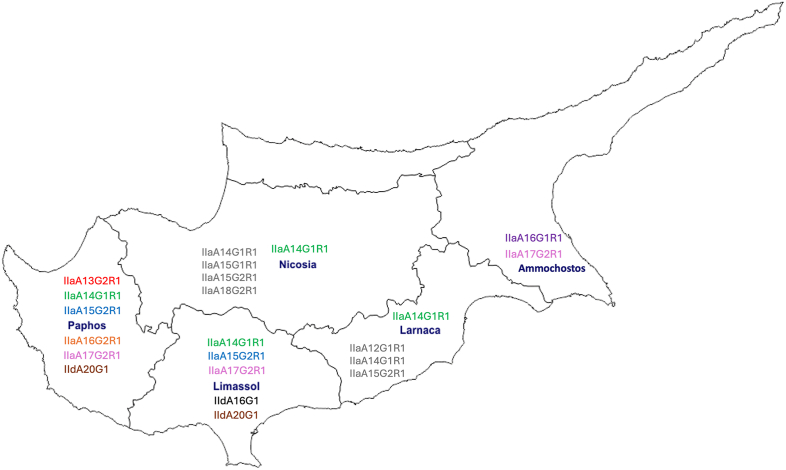
Geographical distribution of *Cryptosporidium parvum* subtypes identified in Cypriot dairy farms. Map of Cyprus showing the five districts where sampling was conducted. Each district is annotated with the *C. parvum gp60* subtypes detected in the present study. Subtypes shown in *grey* represent those previously reported by Hoque et al. (2022), while all other colours indicate subtypes identified herein. The figure highlights both the geographical spread and subtype diversity across the island, with Paphos and Limassol showing the greatest diversity.

Of the 18 farms sampled, 16 (88.9%) harboured *C. parvum* subtypes. Eight farms were found to contain one subtype, two farms contained two, three farms contained three, and one farm harboured four distinct subtypes. The most prevalent subtype was IIaA14G1R1, detected in 9 out of 18 farms (50.0%), and present in all districts except Ammochostos. It was also the only subtype identified in Nicosia and Larnaca. The second most widely distributed subtype, IIaA17G2R1, was detected in 7 out of 18 farms in Limassol, Ammochostos and Paphos. Subtypes IIaA15G2R1, IIdA16G1 and IIdA20G1 were each found in 2 out of 18 farms; IIaA15G2R1 in Limassol and Paphos, IIdA16G1 in Limassol and IIdA20G1 in both Limassol and Paphos. Three subtypes, IIaA13G2R1, IIaA16G1R1 and IIaA16G2R1, were all found in a single farm (1/18) each in Paphos, Ammochostos and Paphos, respectively (Table 3). In total, six subtypes were identified in Paphos, five in Limassol, two in Ammochostos and one each in Nicosia and Larnaca. These subtypes, along with the ones from the study by Hoque et al. (2022), were mapped on a Cyprus map (Fig. 4). The expected counts were greater than zero, indicating that the chi-square test was appropriate to use. The test revealed a highly significant (*P* = 1 × 10^−15^) association between district and *C. parvum* subtype (*χ*^2^ = 85.09, *df* = 8, *P* < 0.001). Of the 122 samples amplified for the *gp60* gene and sequenced, the majority (52.5%, 64/122) were from diarrhoeic calves. Twenty-six of the diarrhoeic samples were marked as Grade 3, 22 as Grade 1 and 16 as Grade 2. Among the subtypes identified, 83.3% (10/12) of IIdA16G1 samples were diarrhoeic, followed by 78.3% (36/46) of IIaA14G1R1 samples, 66.7% (2/3) of IIaA16G1R1, 50.0% (1/2) of IIaA15G2R1, 40.0% (2/5) of IIdA20G1, 24.5% (12/49) of IIaA17G2R1, and 0% (0/1) of both IIaA13G2R1 and IIaA16G2R1.

Of the 122 *gp60* sequence positive samples (23.6%, 122/517), 35.6% were from calves (93/261) and 11.3% from dams (29/256) (Table 2). There were 21 positive pairs (dam and her calf) of *C. parvum* compared to eight positive dams with negative calves and 72 positive calves with negative dams. Dam and calves shared the same subtype in 12 of the 21 positive pairs. Eleven of these were subtype IIaA17G2R1 (1 pair in Ammochostos, 5 in Limassol and 5 in Paphos) and one was subtype IIaA14G1R1 (in Paphos). Close inspection of the sequences revealed that no pair was 100% identical, with two pairs differing by a single polymorphism.

### 3.5 Association of *Cryptosporidium* subtypes with diarrhoea

**Fig. 5:**
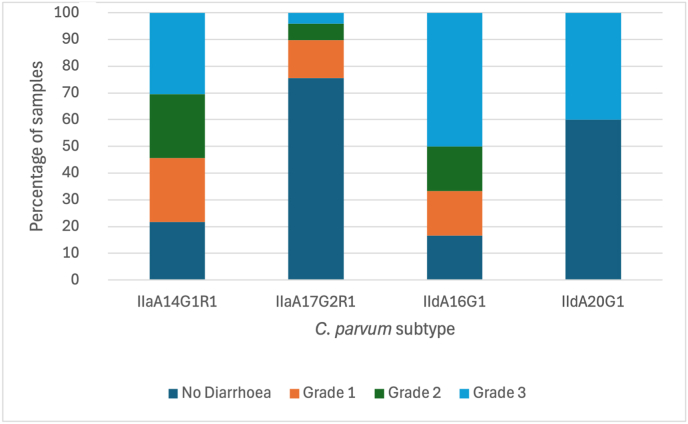
Distribution of diarrhoea status by *Cryptosporidium parvum gp60* subtype. Stacked bar charts depicting the percentage of samples with no diarrhoea (*dark blue*), or diarrhoea Grade 1 (mild; *orange*), 2 (moderate; *green*), or 3 (severe; *light blue*), across *C. parvum gp60* subtypes IIaA14G1R1, IIaA17G2R1, IIdA16G1, and IIdA20G1.

For subtype IIaA14G1R1, 78.3% (36/46) of cases were diarrhoeic and were broadly distributed across all diarrhoea grades, showing the highest frequency of Grade 3 diarrhoea (38.9%, 14/36) (Fig. 5). In contrast, IIaA17G2R1 was predominantly found in non-diarrhoeic calves (75.5%, 37/49). Subtype IIdA16G1 displayed the strongest association with severe diarrhoea, with 83.3% (10/12) of samples being diarrhoeic and 50.0% (6/12) of infected calves presenting with Grade 3 symptoms. The global chi-square test showed highly significant differences in diarrhoea status among *C. parvum* subtypes (*χ*^2^ = 37.45, *df* = 6, *P* < 0.0001). Pairwise chi-square tests showed highly significant differences between IIaA14G1R1 and IIaA17G2R1 (*χ*^2^ = 29.91, *df* = 3, *P* < 0.0001) and between IIaA17G2R1 and IIdA16G1 (*χ*^2^ = 22.06, *df* = 3, *P* < 0.0001). Odds ratio analysis indicated that diarrhoea was significantly more likely in calves with subtype IIaA14G1R1 compared to IIaA17G2R1 (OR = 0.09, Fisher’s exact test, *P <* 0.0001) and in those with IIdA16G1 compared to IIaA17G2R1 (OR = 15.42, Fisher’s exact test, *P* < 0.001).

The SSU rRNA and *gp60* gene sequences have been deposited in GenBank under the accession numbers PV789728-PV789736 and PV808737-PV808858, respectively.

## 4 Discussion

This study determined *Cryptosporidium* spp. infections in dairy cow farms from the five districts of Cyprus, utilising stool samples from our previously established biobank (Mavrides et al., 2024). Of the studied farms, 89% were positive for *Cryptosporidium* spp., matching previous studies in the country and in Europe (Hoque et al., 2022, 2023; Del Río et al., 2025). The overall occurrence of *Cryptosporidium* spp. in calves was 39.5%, aligning with the findings of a previous study in Cyprus (Hoque et al., 2022). Similar studies in the Mediterranean region using molecular methods reported prevalence rates ranging from 16.7% to 38.8% (Díaz et al., 2018, 2021; Kabir et al., 2020; Yildirim et al., 2020), while studies conducted elsewhere ranged from 11.9% to 47.1% (Santoro et al., 2019; Pinto et al., 2021; Zhao et al., 2023; Buchanan et al., 2025). Nonetheless, the prevalence of *Cryptosporidium* spp. in Cyprus is at the higher end of the range of what is typically detected in European countries, a finding that was also reported in calves from the Canary Islands (Del Río et al., 2025). Since islands constitute isolated areas of limited land space, this might hint at presence of island-specific conditions that facilitate transmission. Additionally, some studies focused only on diarrheic animals (Kabir et al., 2020; Yildirim et al., 2020), but as asymptomatic calves can also shed oocysts, differences in sampling strategies may also influence reported positivity rates (Thomson et al., 2017). Finally, variations in husbandry practices, geographical location, climate, or detection methods used could also contribute to the differences in rates of *Cryptosporidium* spp. between regions (Roblin et al., 2023).

*Cryptosporidium parvum* was present in all but two farms and all districts, with a few notable variations. More specifically, Limassol and Paphos had higher positivity rates for the parasite. This may be related to micro-climatic conditions, as these districts experience higher humidity levels, which have been associated with increased *Cryptosporidium* spp. incidence (Ikiroma and Pollock, 2021). Variations in *C. parvu*m occurrence rates between farms (ranging from 2.5% to 39.0%) may also reflect seasonal influences, as increased temperature and precipitation are predictive of higher incidence (Jagai et al., 2009; Szonyi et al., 2010). As sampling was conducted over the course of a year, such seasonal variability may have influenced the detection rates of the parasite between farms. Additional factors, such as differences in herd size, animal husbandry and management practices, are also likely to have contributed to this variation (Trotz-Williams et al., 2008; Abdullah et al., 2019). Further research into the role of both environmental and management-related factors is needed to inform effective control strategies of *Cryptosporidium* and its spread (Szonyi et al., 2010).

The study utilised samples from dams and their calves three to seven days post-parturition. Of the *C. parvum-*positive dams, 72.4% (21/29) had positive corresponding calves, with 12 pairs sharing the same subtype. These were all IIaA17G2R1 except one pair, which was IIaA14G1R1. While there is some debate about whether calves become infected with *C. parvum* at or shortly after birth, it is well established that the parasite is not vertically transmitted (Faubert and Litvinsky, 2000; Silverlås et al., 2010; Pinto et al., 2021; Hoque et al., 2023). A longitudinal study demonstrated negligible subtype overlap between dams and calves, suggesting adult cattle are unlikely to be the primary source of calf *C. parvum* infection (Thomson et al., 2019). This finding is also supported here, whereby none of the dam calf pairs shared identical subtype sequences. Nevertheless, adult cattle may still shed large quantities of oocysts into the environment due to their higher volume of faeces, so they cannot be entirely ruled out as a potential source. The ambiguity surrounding the role of adult cattle in *C. parvum* transmission highlights the need to revisit this question. Farm-level husbandry practices, such as the timing of the calf from the dam separation, use of mixed calving pens, mixing of calves post-parturition and of different age groups, are likely to influence transmission dynamics of *C. parvum* (Barrington et al., 2002; Becher et al., 2004; Bartley et al., 2023). Further research investigating these practices in cattle farming in Cyprus may help clarify their role in parasite prevalence and transmission.

Approximately half of the *C. parvum*-positive samples were from non-diarrhoeic calves, consistent with previous studies. This adds to the evidence that parasite presence is not always associated with diarrhoea, with host- and environmental-related factors influencing the outcome of infection (Naciri et al., 1999; Ouakli et al., 2018; Thomson et al., 2019; Díaz et al., 2021). Hence, asymptomatic calves may still shed oocysts, contributing to environmental contamination and potentially acting as reservoirs for transmission to humans (Díaz et al., 2010).

Eight distinct subtypes of *C. parvum* were identified in this study, belonging to the families IIa and IId. Family IIa is a well-documented zoonotic family exceptionally prevalent in pre-weaned calves worldwide (Buchanan et al., 2025). While most subtypes were present in both diarrheic and non-diarrheic animals, certain subtypes – specifically, IIdA16G1 and IIaA14G1R1 – were more frequently associated with symptomatic hosts (Kabir et al., 2020; Jang et al., 2021). These subtypes have been previously linked to diarrhoea in calves and small ruminants. In contrast, subtype IIaA17G2R1 was detected more frequently in non-diarrheic hosts, and this association was confirmed statistically by odds ratio analysis, which also indicated that IIdA16G1 and IIaA14G1R1 were strongly associated with diarrheic calves. Hence, these findings indicate that there is subtype-specific variation in pathogenicity.

Subtypes of family IId are primarily found in sheep and goats but are also highly prevalent in cattle in the Middle East and China, and were detected in parts of southern Europe (Quilez et al., 2008; Ryan et al., 2014; Feng and Xiao, 2017; Xiao and Feng, 2017; Del Río et al., 2025). To date, this family has not been previously detected in Cypriot cattle. Several IId subtypes have been linked to diarrhoea. Specifically, IIdA20G1 caused outbreaks with significant mortality in neonatal calves in China (Li et al., 2022). Herein, IIdA16G1 was strongly associated with diarrheic calves. Several studies have documented cross-species transmission of IId subtypes, particularly in settings where animals of different species are housed in close contact (Feng and Xiao, 2017; Del Río et al., 2025). This may have been the case in Cyprus, where mixed cattle and sheep/goat farming is sometimes practised. Notably, the IId family was not detected in two earlier small-scale studies of sheep and goats on the island (Schou et al., 2022; Hasapis et al., 2024). Further research is needed to determine the presence of this family in Cypriot ruminants to better understand the transmission dynamics and potential emergence of this subtype on the island.

In the previous study (Hoque et al., 2022), 10 farms were sampled in Nicosia (*n* = 8) and Larnaca (*n* = 2), with only one farm overlapping with the present study. Hoque et al. (2022) detected five *C. parvum* subtypes (IIaA12G1R1, IIaA14G1R1, IIaA15G1R1, IIaA15G2R1, and IIaA18G2R1), all belonging to family IIa. Herein, eight subtypes were detected, only two of which overlapped with those previously reported (IIaA14G1R1 and IIaA15G2R1), while the remaining subtypes had not been previously detected in cattle on the island. In terms of districts, Hoque et al. (2022) detected four subtypes in Nicosia and three in Larnaca; however, here only IIaA14G1R1 was detected in both districts. Incidentally, this same subtype was the most geographically widespread in both studies, most abundant in the previous study and second most abundant in the present study, being present in all districts except one, indicating this subtype’s stability over time. Our results suggest a geographical clustering of subtypes, with some being more abundant in some districts and absent in others. This geographical distribution pattern was statistically confirmed, though further studies are needed to validate these associations. Even though there was little overlap in terms of farms sampled in the two studies, the changes in geographical distribution of *C. parvum* subtypes in dairy cattle in Cyprus over time add to the epidemiology of *C. parvum*. Such temporal shifts in *C. parvum* subtype distribution at a country level have been previously noted (Kaupke and Rzeżutka, 2022). Alternatively, it is possible that the previously identified subtypes were simply not detected or were not present in the farms sampled. Additional longitudinal sampling is required to determine whether these changes indeed reflect true shifts in subtype dynamics and what these might mean. It would be interesting to see whether IIaA14G1R1 continues to expand across districts.

All the subtypes detected here have been linked to human infections globally, a proportion of which have been responsible for severe human cryptosporidiosis outbreaks, highlighting their significant zoonotic potential and risk to public health (CDC, 2011; Hijjawi et al., 2017; Robertson et al., 2019; Garcia-R et al., 2020; Suominen et al., 2023). For instance, IIaA15G2R1 has caused most of the acute clinical infections in humans and is a frequently detected subtype worldwide (Xiao, 2010; Feng et al., 2018; Chen et al., 2023). Given the number of zoonotic subtypes circulating on the island, its small size and the proximity of farms to residential areas, it is indeed surprising that an outbreak of cryptosporidiosis has yet to be reported in Cyprus. Cryptosporidiosis is not a notifiable disease in humans in Cyprus, and therefore, there is no compulsory national reporting system in place. As a result, no official data on the annual number of human cases is published, and Cyprus did not report any cryptosporidiosis cases to the ECDC between 2008 and 2018. To date, no formal outbreaks have been documented on the island, nor is there evidence of systematic epidemiological investigations related to *Cryptosporidium* spp. The absence of routine diagnostic protocols and laboratory-based case reporting suggests that cases may go undetected or not investigated, unless identified in private healthcare settings. Notably, in 2022, the UK *Cryptosporidium* Reference Unit reported that Cyprus was the second most common travel destination associated with cryptosporidiosis cases among genotyped specimens from England and Wales (Public Health Wales Microbiology, 2023). Further epidemiological studies that would include the human population of the island, international travellers and the environment would be necessary to clarify transmission dynamics under the One Health umbrella.

This study has certain limitations. The cross-sectional design limited the ability to assess the time course of infection and to fully capture the temporal dynamics and diversity of *Cryptosporidium* species and *C. parvum gp60* subtypes. Nonetheless, longitudinal follow-up of individual animals was not feasible due to the dynamic nature of animal management within farms, where animals are frequently relocated, making tracking and re-sampling difficult. Additionally, subtyping was only performed for *C. parvum* at the *gp60* locus; for other species, such as *C. bovis* and *C. ryanae*, only SSU rRNA gene sequencing was carried out as *gp60* amplification tools for these species were not used in this study. Finally, further associations between infection and farm-level husbandry practices were not possible as such information was not collected.

## 5 Conclusions

This study offers the first detailed molecular insight into *Cryptosporidium* spp. infections in dairy cattle in Cyprus, identifying multiple species and a diverse range of *C. parvum gp60* subtypes, including two IId subtypes not previously reported in the country. The occurrence of specific subtypes with either severe diarrhoea or asymptomatic infection adds to our understanding of their clinical relevance. Given the zoonotic potential of all identified subtypes, these findings underscore the importance of adopting a One Health approach. Detailed investigations at the subtype level using Multilocus Variable-Number Tandem-Repeat Analysis (MLVA) or Multilocus Sequence Typing (MLST) are needed in Cyprus and elsewhere to identify potential outbreak-associated variants and to better understand subtype circulation. Future research focusing on simultaneously acquiring animal, human and environmental data, alongside longitudinal studies and farm-level management assessments, would be valuable in interpreting parasite prevalence and understanding transmission dynamics.

## Ethical approval

Farm owners provided informed consent for participation in the study.

## CRediT authorship contribution statement

**Daphne E. Mavrides:** Methodology, Validation, Formal analysis, Investigation, Writing - original draft. **Maria Liapi:** Investigation, Project administration. **Stavros Malas:** Writing - review & editing, Visualization, Supervision, Project administration. **Anastasios D. Tsaousis:** Conceptualization, Validation, Resources, Writing - review & editing, Visualization, Supervision, Project administration, Funding acquisition. **Eleni Gentekaki:** Conceptualization, Validation, Resources, Data curation, Writing - review & editing, Supervision, Project administration, Funding acquisition.

## Funding

The project is funded by the 1HEALTH-GUTBIOME seed grant awarded by the 10.13039/501100019592University of Nicosia to EG.

## Declaration of competing interests

The authors declare that they have no known competing financial interests or personal relationships that could have appeared to influence the work reported in this paper.

## Data Availability

The data supporting the conclusions of this article are included within the article. Raw data are available from the corresponding author upon request. The newly generated SSU rRNA and *gp60* gene sequences have been deposited in the GenBank database under the accession numbers PV789728-PV789736 and PV808737-PV808858, respectively.

## References

[bib1] Abdullah D.A., Ola-Fadunsin S., Ruviniyia K., Gimba F.I., Chandrawathani P., Lim Y.A.L. (2019). Molecular detection and epidemiological risk factors associated with *Cryptosporidium* infection among cattle in Peninsular Malaysia. Food Waterborne Parasitol..

[bib2] Alves M., Xiao L., Antunes F., Matos O. (2006). Distribution of *Cryptosporidium* subtypes in humans and domestic and wild ruminants in Portugal. Parasitol. Res..

[bib3] Alves M., Xiao L., Sulaiman I., Lal A.A., Matos O., Antunes F. (2003). Subgenotype analysis of *Cryptosporidium* isolates from humans, cattle, and zoo ruminants in Portugal. J. Clin. Microbiol..

[bib4] Amer S., Honma H., Ikarashi M., Tada C., Fukuda Y., Suyama Y., Nakai Y. (2010). *Cryptosporidium* genotypes and subtypes in dairy calves in Egypt. Vet. Parasitol..

[bib5] Arsenopoulos K., Theodoridis A., Papadopoulos E. (2017). Effect of colostrum quantity and quality on neonatal calf diarrhoea due to *Cryptosporidium* spp. infection. Comp. Immunol. Microbiol. Infect. Dis..

[bib6] Avendaño C., Ramo A., Vergara-Castiblanco C., Sánchez-Acedo C., Quílez J. (2018). Genetic uniqueness of *Cryptosporidium parvum* from dairy calves in Colombia. Parasitol. Res..

[bib7] Ayres Hutter J., Dion R., Irace-Cima A., Fiset M., Guy R., Dixon B. (2020). *Cryptosporidium* spp.: human incidence, molecular characterization and associated exposures in Québec, Canada (2016–2017). PLoS One.

[bib8] Baroudi D., Khelef D., Hakem A., Abdelaziz A., Chen X., Lysen C. (2017). Molecular characterization of zoonotic pathogens *Cryptosporidium* spp., *Giardia duodenalis* and *Enterocytozoon bieneusi* in calves in Algeria. Vet. Parasitol. Reg. Stud. Rep..

[bib9] Barrington G.M., Gay J.M., Evermann J.F. (2002). Biosecurity for neonatal gastrointestinal diseases. Vet. Clin. North Am. Food Anim. Pract..

[bib10] Bartley P.M., Standar J.H., Katzer F. (2023). Genetic characterisation of *Cryptosporidium parvum* in dairy cattle and calves during the early stages of a calving season. Curr. Res. Parasitol. Vector Borne Dis..

[bib11] Becher K.A., Robertson I.D., Fraser D.M., Palmer D.G., Thompson R.C.A. (2004). Molecular epidemiology of *Giardia* and *Cryptosporidium* infections in dairy calves originating from three sources in Western Australia. Vet. Parasitol..

[bib12] Bordes L., Houert P., Costa D., Favennec L., Vial-Novella C., Fidelle F. (2020). Asymptomatic *Cryptosporidium* infections in ewes and lambs are a source of environmental contamination with zoonotic genotypes of *Cryptosporidium parvum*. Parasite.

[bib13] Broglia A., Reckinger S., Cacció S.M., Nöckler K. (2008). Distribution of *Cryptosporidium parvum* subtypes in calves in Germany. Vet. Parasitol..

[bib14] Brook E.J., Anthony Hart C., French N.P., Christley R.M. (2009). Molecular epidemiology of *Cryptosporidium* subtypes in cattle in England. Vet. J..

[bib15] Buchanan R., Wieckowski P., Matechou E., Katzer F., Tsaousis A.D., Farré M. (2025). Global prevalence of *Cryptosporidium* infections in cattle: a meta-analysis. Curr. Res. Parasitol. Vector Borne Dis..

[bib16] CDC (2011). https://www.cdc.gov/mmwr/preview/mmwrhtml/mm6027a3.htm#:%7E:text=A%20total%20of%2046%20cases,parvum%20subtype%2C%20IIaA17G2R1.

[bib17] Chalmers R.M., Batt C.A., Lou Tortorello M. (2014). Encyclopedia of Food Microbiology.

[bib18] Chalmers R.M., Robinson G., Elwin K., Elson R. (2019). Analysis of the *Cryptosporidium* spp. and *gp60* subtypes linked to human outbreaks of cryptosporidiosis in England and Wales, 2009 to 2017. Parasites Vectors.

[bib19] Chen Y., Huang J., Qin H., Wang L., Li J., Zhang L. (2023). *Cryptosporidium parvum* and *gp60* genotype prevalence in dairy calves worldwide: a systematic review and meta-analysis. Acta Trop..

[bib20] de Graaf D.C., Vanopdenbosch E., Ortega-Mora L., Abbassi H., Peeters J.E. (1999). A review of the importance of cryptosporidiosis in farm animals. Int. J. Parasitol..

[bib21] Del Río M.C., Martín S., Quílez J., Vergara-Castiblanco C., Molina J.M., Ferrer O. (2025). Molecular analysis of cryptosporidiosis on cattle farms in Gran Canaria, Canary Islands (Spain). Int. J. Vet. Sci. Med..

[bib22] Díaz P., Navarro E., Remesar S., García-Dios D., Martínez-Calabuig N., Prieto A. (2021). The age-related *Cryptosporidium* species distribution in asymptomatic cattle from northwestern Spain. Animals.

[bib23] Díaz P., Qulez J., Chalmers R.M., Panadero R., López C., Sánchez-Acedo C. (2010). Genotype and subtype analysis of *Cryptosporidium* isolates from calves and lambs in Galicia (NW Spain). Parasitology.

[bib24] Díaz P., Varcasia A., Pipia A.P., Tamponi C., Sanna G., Prieto A. (2018). Molecular characterisation and risk factor analysis of *Cryptosporidium* spp. in calves from Italy. Parasitol. Res..

[bib25] Dixon B.R., Motarjemi Y. (2014). Encyclopedia of Food Safety.

[bib26] Faubert G.M., Litvinsky Y. (2000). Natural transmission of *Cryptosporidium parvum* between dams and calves on a dairy farm. J. Parasitol..

[bib27] Feng Y., Ryan U.M., Xiao L. (2018). Genetic diversity and population structure of *Cryptosporidium*. Trends Parasitol..

[bib28] Feng Y., Xiao L. (2017). Molecular epidemiology of cryptosporidiosis in China. Front. Microbiol..

[bib29] Garcia-R J., Pita A.B., Velathanthiri N., French N.P., Hayman D.T.S. (2020). Species and genotypes causing human cryptosporidiosis in New Zealand. Parasitol. Res..

[bib30] Giadinis N.D., Symeoudakis S., Papadopoulos E., Lafi S.Q., Karatzias H. (2012). Comparison of two techniques for diagnosis of cryptosporidiosis in diarrhoeic goat kids and lambs in Cyprus. Trop. Anim. Health Prod..

[bib31] Gong C., Cao X.-F., Deng L., Li W., Huang X.-M., Lan J.-C. (2017). Epidemiology of *Cryptosporidium* infection in cattle in China: a review. Parasite.

[bib32] Hasapis K.A., Charalambidou I., Schou C., O'Dowd Phanis C., Kazamia S., Kassinis N. (2024). First detection of *Cryptosporidium parvum* in the endemic Cyprus mouflon (*Ovis gmelini ophion*). Acta Parasitol..

[bib33] Hasapis K.A., Charalambidou I., Tsouma E., Sotiriadi K., Kassinis N., Schou C., Karanis P. (2023). First detection of *Cryptosporidium proventriculi* from wild birds in Cyprus. Parasitol. Res..

[bib34] Hatam-Nahavandi K., Ahmadpour E., Carmena D., Spotin A., Bangoura B., Xiao L. (2019). *Cryptosporidium* infections in terrestrial ungulates with focus on livestock: a systematic review and meta-analysis. Parasites Vectors.

[bib35] Hijjawi N., Zahedi A., Kazaleh M., Ryan U. (2017). Prevalence of *Cryptosporidium* species and subtypes in paediatric oncology and non-oncology patients with diarrhoea in Jordan. Infect. Genet. Evol..

[bib36] Hoque S., Mavrides D.E., Pinto P., Costas S., Begum N., Azevedo-Ribeiro C. (2022). High occurrence of zoonotic subtypes of *Cryptosporidium parvum* in Cypriot dairy farms. Microorganisms.

[bib37] Hoque S., Pinto P., Ribeiro C.A., Canniere E., Daandels Y., Dellevoet M. (2023). Follow-up investigation into *Cryptosporidium* prevalence and transmission in Western European dairy farms. Vet. Parasitol..

[bib38] Ikiroma I.A., Pollock K.G. (2021). Influence of weather and climate on cryptosporidiosis - a review. Zoonoses Publ. Health.

[bib39] Jagai J.S., Castronovo D.A., Monchak J., Naumova E.N. (2009). Seasonality of cryptosporidiosis: a meta-analysis approach. Environ. Res..

[bib40] Jang D.-H., Cho H.-C., Shin S.-U., Kim E.-M., Park Y.-J., Hwang S. (2021). Prevalence and distribution pattern of *Cryptosporidium* spp. among pre-weaned diarrheic calves in the Republic of Korea. PLoS One.

[bib41] Kabir M.H.B., Ceylan O., Ceylan C., Shehata A.A., Bando H., Essa M.I. (2020). Molecular detection of genotypes and subtypes of *Cryptosporidium* infection in diarrheic calves, lambs, and goat kids from Turkey. Parasitol. Int..

[bib42] Kaupke A., Rzeżutka A. (2022). Population genetics of *Cryptosporidium parvum* subtypes in cattle in Poland: the geographical change of strain prevalence and circulation over time. BMC Vet. Res..

[bib43] Khan S.M., Debnath C., Pramanik A.K., Xiao L., Nozaki T., Ganguly S. (2010). Molecular characterization and assessment of zoonotic transmission of *Cryptosporidium* from dairy cattle in West Bengal, India. Vet. Parasitol..

[bib44] Li M., Yang F., Hou T., Gong X., Li N., Sibley L.D. (2024). Variant surface protein GP60 contributes to host infectivity of *Cryptosporidium parvum*. Commun. Biol..

[bib45] Li N., Zhao W., Song S., Ye H., Chu W., Guo Y. (2022). Diarrhoea outbreak caused by coinfections of *Cryptosporidium parvum* subtype IIdA20G1 and rotavirus in pre-weaned dairy calves. Transbound. Emerg. Dis..

[bib46] Mavrides D.E., Liapi M., Ierodiakonou D., Pipis C., Malas S., Gentekaki E., Tsaousis A.D. (2024). The cow GUTBIOME CY study: investigating the composition of the cattle gut microbiome in health and infectious disease transmission in Cyprus. BMC Vet. Res..

[bib47] Naciri M., Paul Lefay M., Mancassola R., Poirier P., Chermette R. (1999). Role of *Cryptosporidium parvum* as a pathogen in neonatal diarrhoea complex in suckling and dairy calves in France. Vet. Parasitol..

[bib48] Nydam D.V., Wade S.E., Schaaf S.L., Mohammed H.O. (2001). Number of *Cryptosporidium parvum* oocysts or *Giardia* spp. cysts shed by dairy calves after natural infection. Am. J. Vet. Res..

[bib49] Ouakli N., Belkhiri A., de Lucio A., Köster P.C., Djoudi M., Dadda A. (2018). *Cryptosporidium*-associated diarrhoea in neonatal calves in Algeria. Vet. Parasitol. Reg. Stud. Rep..

[bib50] Pinto P., Ribeiro C.A., Hoque S., Hammouma O., Leruste H., Détriché S. (2021). Cross-border investigations on the prevalence and transmission dynamics of *Cryptosporidium* species in dairy cattle farms in western mainland Europe. Microorganisms.

[bib51] Public Health Wales Microbiology (2023). https://phw.nhs.wales/services-and-teams/cryptosporidium-reference-unit/annual-report-of-referrals-and-cryptosporidium-genotyping-england-and-wales-2022/.

[bib52] Quilez J., Torres E., Chalmers R.M., Robinson G., Del Cacho E., Sanchez-Acedo C. (2008). *Cryptosporidium* species and subtype analysis from dairy calves in Spain. Parasitology.

[bib53] Robertson L.J., Temesgen T.T., Tysnes K.R., Eikås J.E. (2019). An apple a day: an outbreak of cryptosporidiosis in Norway associated with self-pressed apple juice. Epidemiol. Infect..

[bib54] Robinson G., Chalmers R.M., Elwin K., Guy R.A., Bessonov K., Troell K., Xiao L. (2025). Deciphering a cryptic minefield: a guide to *Cryptosporidium* gp60 subtyping. Curr. Res. Parasitol. Vector Borne Dis..

[bib55] Roblin M., Canniere E., Barbier A., Daandels Y., Dellevoet-Groenewegen M., Pinto P. (2023). Study of the economic impact of cryptosporidiosis in calves after implementing good practices to manage the disease on dairy farms in Belgium, France, and the Netherlands. Curr. Res. Parasitol. Vector Borne Dis..

[bib56] Ryan U., Fayer R., Xiao L. (2014). *Cryptosporidium* species in humans and animals: current understanding and research needs. Parasitology.

[bib57] Santín M., Trout J.M., Fayer R. (2008). A longitudinal study of cryptosporidiosis in dairy cattle from birth to 2 years of age. Vet. Parasitol..

[bib58] Santín M., Trout J.M., Xiao L., Zhou L., Greiner E., Fayer R. (2004). Prevalence and age-related variation of *Cryptosporidium* species and genotypes in dairy calves. Vet. Parasitol..

[bib59] Santoro A., Dorbek-Kolin E., Jeremejeva J., Tummeleht L., Orro T., Jokelainen P., Lassen B. (2019). Molecular epidemiology of *Cryptosporidium* spp. in calves in Estonia: high prevalence of *Cryptosporidium parvum* shedding and 10 subtypes identified. Parasitology.

[bib60] Schou C., Hasapis K., Karanis P. (2022). Molecular identification of *Cryptosporidium* species from domestic ruminants and wild reptiles in Cyprus. Parasitol. Res..

[bib61] Shaw H.J., Innes E.A., Morrison L.J., Katzer F., Wells B. (2020). Long-term production effects of clinical cryptosporidiosis in neonatal calves. Int. J. Parasitol..

[bib62] Silverlås C., Näslund K., Björkman C., Mattsson J.G. (2010). Molecular characterisation of *Cryptosporidium* isolates from Swedish dairy cattle in relation to age, diarrhoea and region. Vet. Parasitol..

[bib63] Suler D., Mullins D., Rudge T., Ashurst J. (2016). *Cryptosporidium parvum* infection following contact with livestock. North Am. J. Med. Sci..

[bib64] Suominen K.A., Björkstrand M., Ollgren J., Autio T.J., Rimhanen-Finne R. (2023). Cryptosporidiosis in Finland is predominantly of domestic origin: investigation of increased reporting, 1995–2020. Infect. Dis..

[bib65] Szonyi B., Bordonaro R., Wade S.E., Mohammed H.O. (2010). Seasonal variation in the prevalence and molecular epidemiology of *Cryptosporidium* infection in dairy cattle in the New York City Watershed. Parasitol. Res..

[bib66] Thomson S., Hamilton C.A., Hope J.C., Katzer F., Mabbott N.A., Morrison L.J., Innes E.A. (2017). Bovine cryptosporidiosis: impact, host-parasite interaction and control strategies. Vet. Res..

[bib67] Thomson S., Innes E.A., Jonsson N.N., Katzer F. (2019). Shedding of *Cryptosporidium* in calves and dams: evidence of re-infection and shedding of different *gp60* subtypes. Parasitology.

[bib68] Trotz-Williams L., Martin S.W., Leslie K.E., Duffield T., Nydam D.V., Peregrine A.S. (2008). Association between management practices and within-herd prevalence of *Cryptosporidium parvum* shedding on dairy farms in southern Ontario. Prev. Vet. Med..

[bib69] Vermeulen L.C., Hengel M.V., Kroeze C., Medema G., Spanier J.E., Vliet M.T.H.V., Hofstra N. (2019). *Cryptosporidium* concentrations in rivers worldwide. Water Res..

[bib70] Xiao L. (2010). Molecular epidemiology of cryptosporidiosis: an update. Exp. Parasitol..

[bib71] Xiao L., Feng Y. (2017). Molecular epidemiologic tools for waterborne pathogens *Cryptosporidium* spp. and *Giardia duod*enalis. Food Waterborne Parasitol..

[bib72] Yildirim A., Adanir R., Inci A., Yukari B.A., Duzlu O., Onder Z. (2020). Prevalence and genotyping of bovine *Cryptosporidium* species in the Mediterranean and Central Anatolia region of Turkey. Comp. Immunol. Microbiol. Infect. Dis..

[bib73] Zhao L., Chai H.-L., Wang M.-Y., Zhang Z.-S., Han W.-X., Yang B. (2023). Prevalence and molecular characterization of *Cryptosporidium* spp. in dairy cattle in central Inner Mongolia, northern China. BMC Vet. Res..

[bib74] Ziegler P.E., Santucci F., Lindergard G., Nydam D.V., Wade S.E., Schaaf S.L. (2007). Evaluation of polymerase chain reaction diagnosis of *Cryptosporidium* spp. in dairy cattle and wildlife. Vet. Ther..

